# Three-dimensional tracking using a single-spot rotating point spread function created by a multiring spiral phase plate

**DOI:** 10.1117/1.JBO.27.12.126501

**Published:** 2022-12-29

**Authors:** Keith Bonin, Sudhakar Prasad, Will Caulkins, George Holzwarth, Stephen R. Baker, Pierre-Alexandre Vidi

**Affiliations:** a Wake Forest University, Department of Physics, Winston-Salem, North Carolina, United States; b Atrium Health/Wake Forest Baptist, Comprehensive Cancer Center, Winston-Salem, North Carolina, United States; c University of Minnesota, Department of Physics, Minneapolis, Minnesota, United States; d Wake Forest School of Medicine, Department of Cancer Biology, Winston-Salem, North Carolina, United States; e Institut de Cancérologie de l’Ouest, Angers, France

**Keywords:** diffractive optical elements, microscopy, tracking, imaging, three dimensions, fluorescence

## Abstract

**Significance:**

Three-dimensional (3D) imaging and object tracking is critical for medical and biological research and can be achieved by multifocal imaging with diffractive optical elements (DOEs) converting depth (z) information into a modification of the two-dimensional image. Physical insight into DOE designs will spur this expanding field.

**Aim:**

To precisely track microscopic fluorescent objects in biological systems in 3D with a simple low-cost DOE system.

**Approach:**

We designed a multiring spiral phase plate (SPP) generating a single-spot rotating point spread function (SS-RPSF) in a microscope. Our simple, analytically transparent design process uses Bessel beams to avoid rotational ambiguities and achieve a significant depth range. The SPP was inserted into the Nomarski prism slider of a standard microscope. Performance was evaluated using fluorescent beads and in live cells expressing a fluorescent chromatin marker.

**Results:**

Bead localization precision was <25  nm in the transverse dimensions and ≤70  nm along the axial dimension over an axial range of 6  μm. Higher axial precision (≤50  nm) was achieved over a shallower focal depth of 2.9  μm. 3D diffusion constants of chromatin matched expected values.

**Conclusions:**

Precise 3D localization and tracking can be achieved with a SS-RPSF SPP in a standard microscope with minor modifications.

## 1 Introduction

Some of the most successful methods to localize and track fluorescent emitters in three-dimensions (3D) involve encoding 3D information in a single two-dimensional (2D) image using an engineered point-spread function (EPSF). Early work to develop EPSF-based 3D localization methods in microscopy include rotating PSFs (RPSFs) reported by Piestun’s group,^1^^–^^5^ Moerner’s group,^6^^,^^7^ Prasad,^8^^–^^10^ Ritsch-Marte’s group^11^ and Bouchal’s group,^12^ and nonrotating ones, such as astigmatic,^13^ phase-ramp,^14^ self-bending,^15^ and tetrapod^16^ PSFs. Other 3D localization approaches have used biplane^17^ and multiplane^18^ focusing. Moerner and collaborators used a RPSF to achieve subdiffraction 3D localization of single molecules using both a single-spot corkscrew PSF^6^ and a double-helix PSF^7^ (DH-PSF)—a commercially available two-spot RPSF design, both based on diffractive Laguerre–Gauss (LG) modes. Several applications of the DH-PSF-based localization and tracking have been demonstrated, including particle tracking velocimetry in microfluidics,^19^ visualization of hopping diffusion at interfaces,^20^ imaging of core structures in RNA stress granules,^21^ and improved-signal-to-noise ratio (SNR) imaging when combined with variable-angle light-sheet illumination.^22^^,^^23^ A summary of the DH-PSF and the four-spot tetrapod version are given in a review of single-molecule localization by von Diezmann, et al.^7^ In a relevant recent publication, Gustavsson et al.^24^ reported on a complex 3D tracking microscope system with a custom light sheet illumination subsystem to track chromatin motion in cell nuclei. Their optical apparatus used both the DH-PSF and the tetrapod PSF in a two-channel implementation.

Here we consider a spiral phase plate (SPP) design to perform 3D tracking using a single-spot RPSF (SS-RPSF) that takes advantage of nondiffracting vortex beams and was first proposed by Prasad.^8^^–^^10^ Single-spot approaches offer several advantages over two-spot systems. First, single-spot systems can attain twice the rotation range of a two-spot pattern without confusing spots’ angular positions. Second, emitter densities can theoretically be larger with single spot images than with double spot ones. Third, because the light field for a single emitter is concentrated in one region in the image plane for a single-spot design, optimized designs have the potential for higher SNR than if the same number of photons are distributed over two spots in the image plane. Fourth, it is easy, at least for our design, to visually distinguish when emitters are above or below the focal plane because the corresponding single-spot intensity patterns have opposite helicities above and below the focal plane.

The earliest experimental implementation of Prasad’s analytical design approach was carried out with a low numerical aperture (NA) system (NA=0.75).^11^ Two different polarizations using two different SPP patterns on a single spatial light modulator (SLM) produced images in separate regions of the same camera. The two images of the single spots were then combined to produce two-spot patterns for analysis. Other implementations of this design idea have all been two-spot versions.^12^^,^^25^^–^^27^ Simulations show that a single-spot design could also be used to track space debris in 3D.^28^^,^^29^

This is the first experimental test of a SS-RPSF generating SPP in a high NA microscope. It is also the first experimental test of a seven-annular-zone SPP that maximizes the z-depth range while minimizing the spot size and maintaining the uniformity of the angular rate of rotation (ROR). This design uses (quasi) nondiffracting Bessel beams as the basis set for fields (in contrast to the LG basis set used in other approaches), which leads to the generation of cleaner and simpler SPP designs that can be optimized by means of simple analytical formulas. Key advantages of the SS-RPSF described here are low cost, simplicity, and straightforward compatibility with research-level microscopes, which could accelerate the adoption of 3D imaging by research groups lacking advanced expertise or resources in optics. This contrasts with previously developed 3D imaging systems based on SLMs or phase plates within 4f optical systems, which are more complex to implement and operate. Our SPP is simply inserted into the objective turret nosepiece, in the same position as a Nomarski prism would be placed. In contrast to standard SLM implementations, our optimized SPP design utilizing a fused silica diffractive optical element (DOE) collects all polarizations, thereby maximizing signal-to-noise values. To showcase future bioimaging applications of the SS-RPSF, we applied our SPP design to track chromatin motions in the 3D environment of the mammalian cell nucleus.

## 2 Methods

### 2.1 Spiral Phase Plate

**Fig. 1:**
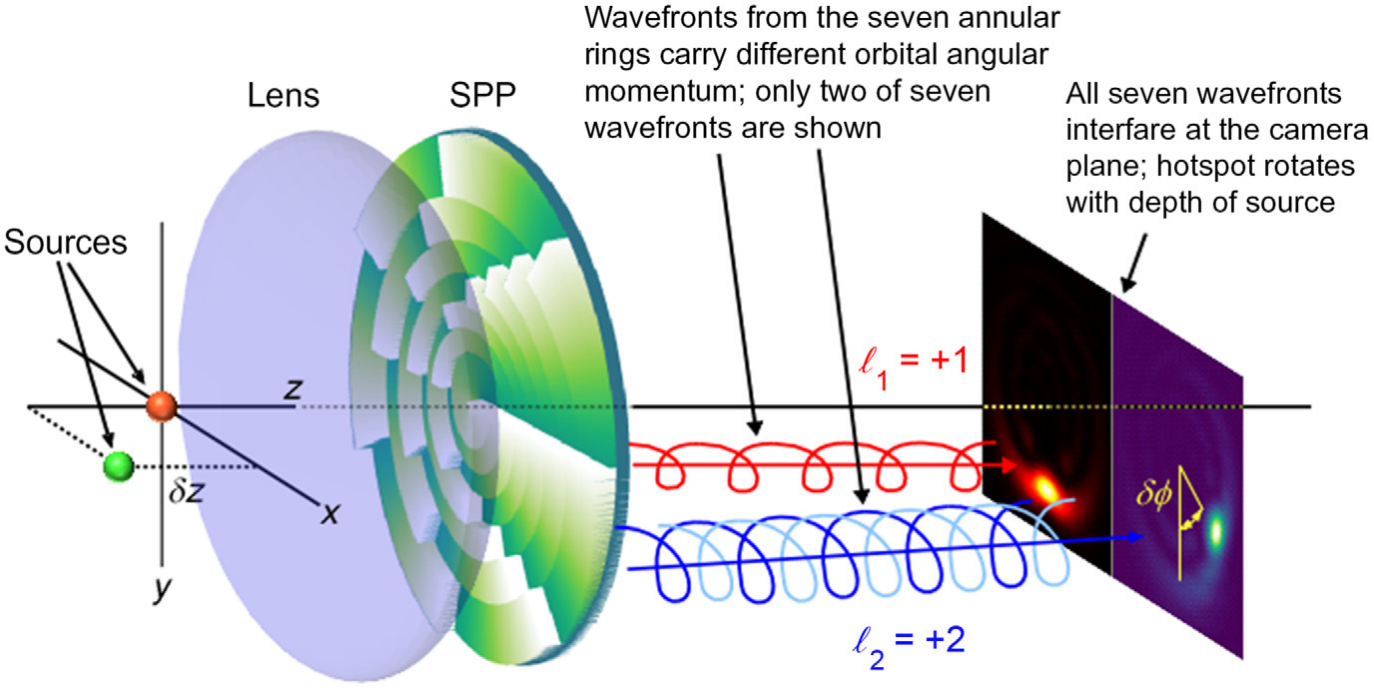
Schematic diagram for encoding 3D source location in 2D images. Two point sources (orange and green) are shown at the left. The orange source is in axial focus. The green source is displaced along x and z with δz<0. The seven zones of the SPP each impart a unique OAM to the imaging wavefront with the OAM eigenvalue of the l’th zone being lℏ. A specific choice of the zone radii allows the seven OAM wavefronts, two of which are shown spiraling, to interfere constructively at the camera plane to create a bright spot that is rotated about the Gaussian image of the source by an angle proportional to the source misfocus (δz). See text for details.

A perspective view of the SPP is shown in green in Fig. 1. The central SPP design idea is to generate a coherent superposition of several nondiffracting Bessel vortex modes using a SPP placed in the aperture of an imaging system. Compared with LG modes,^30^^–^^32^ the Bessel modes^33^^,^^34^ have significantly reduced diffraction as they propagate. In our case, the SPP employs seven concentric annular zones, or rings, of spiral phase retardation with changing orbital angular momentum (OAM) quantum number between the zones. Designing the SPP so that this OAM quantum number increments in regular steps between successive zones and the initial phase discontinuity lines of all zones are azimuthally aligned allows the Bessel beams to interfere constructively and yield a compact image that, for a specific choice of zone radii, rotates uniformly and unidirectionally with changing depth of the light emitter. An approximate analytical expression for the ROR of the emitter image with its depth change may be derived from Eqs. (18) and (19) of Ref. 9 using the latter equation to express the left-hand side of the former in terms of the ratio, l/L, and then setting it equal to the spiral phase, lθ, corresponding to the l’th zone. Differentiating that equality yields the ROR, which may be expressed in terms of the system parameters as

$$\frac{d\theta }{dz}=\frac{2\pi n_{0}}{\lambda_{0}L}(1-\sqrt{1-\frac{{\mathrm{NA}}^{2}}{n_{0}^{2}}}),$$ (1)

where NA is the numerical aperture of the objective that is collecting the light, L is the total number of zones (L=7 here), $\lambda_{0}$ is the wavelength of light in vacuum, and $n_{0}$ is the refractive index of the immersion oil.

The original SPP design described by Prasad^8^ has a number of concentric annular zones, each with a continuous spiral phase ramp. The l’th zone consists of a single azimuthal ramp (vortex) such that the optical phase retardation increases continuously from zero to 2πl radians, whereas the azimuthal angle goes from zero to 2π radians, requiring an optical phase retardation as large as 14π radians, for example, for a seven-zone design. Manufacturing such an SPP to the required precision is not possible with current DOE fabrication technology. This major practical obstacle inherent in the original SPP design was circumvented by Ritsch-Marte’s group^11^ in 2014 by subdividing the l’th annular zone into l subramps, each of which increases in optical phase retardation from 0 to 2π at the design imaging wavelength, like a spiral staircase (Fig. 1). The present paper is a study of the performance of a fused silica DOE using such a modified phase-wrapped design with further modifications included to account for the high NA of modern microscopes.^9^ Our high-NA SPP was fabricated by Vortex Photonics, Planegg, Germany.

### 2.2 Microscope Setup and Image Analysis

**Fig. 2:**
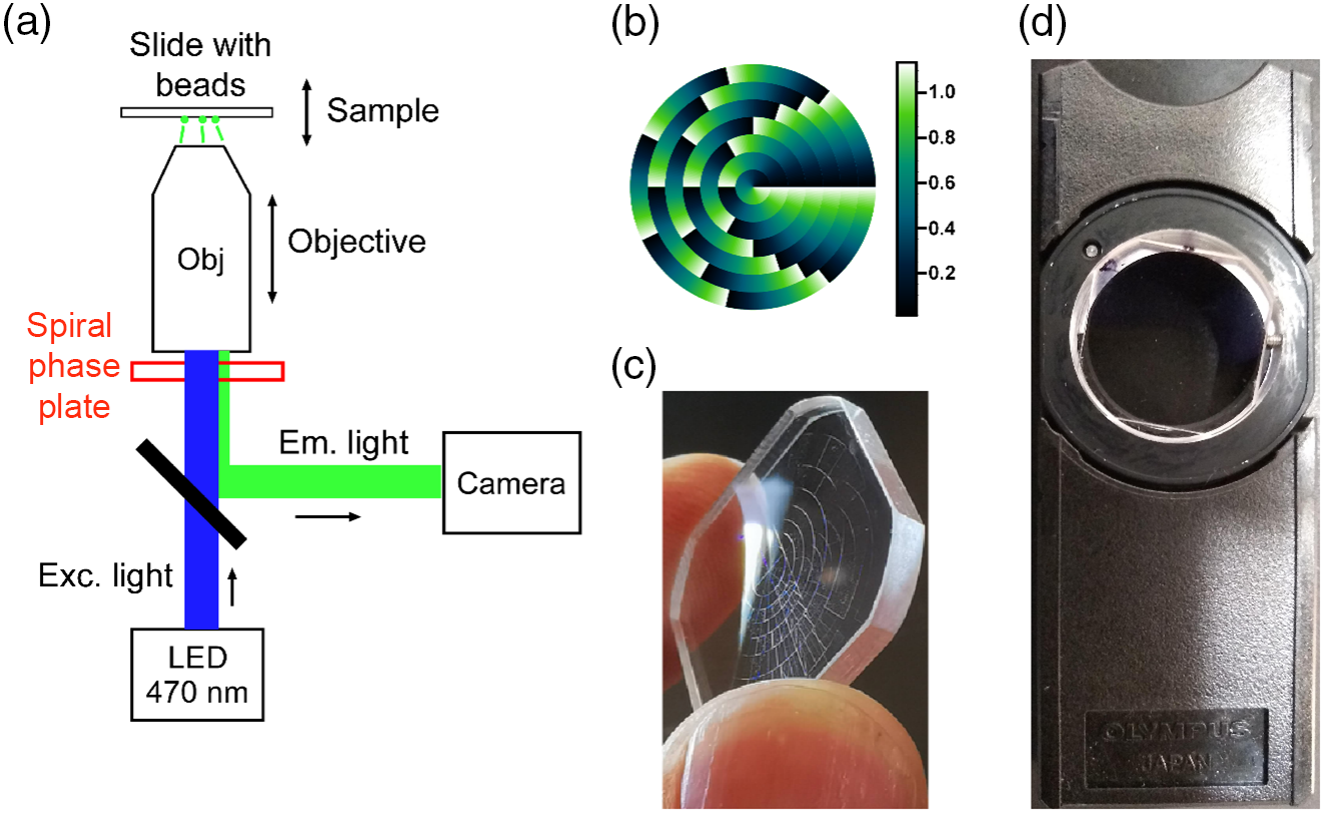
(a) Schematic diagram of the microscope setup, shown for an upright system. (b) SPP structure viewed from above, and falsely colored to show height of structures. Colormap scale is in *μ*m with the height range from 0 to 1.13 *μ*m. (c) Glancing view of actual SPP showing the structure of the surface. (d) SPP mounted in the Nomarski prism slider, for insertion in the microscope nosepiece. (b) and (c) Courtesy of Vortex Photonics, Planegg, Germany.

An Olympus IX-71 microscope was used for the experiments with fluorescent microbeads. The objective had a magnification of 60×, NA = 1.3 and used silicone oil (refractive index = 1.406) as the immersion medium. Images were taken under two scenarios: a fixed sample stage and a moving objective or a moving sample stage and a fixed objective, as shown in Fig. 2(a). In the first scenario (moving objective), movement along the z-axis was controlled using a Physik Instrumente PIFOC objective scanner (P/N PI72Z2CAQ); in the second scenario (moving stage), the z-position of the sample was controlled by a piezoelectric Mad City Labs stage (Model MCL-MOTNZ). In both cases, the relative position between the sample bead plane and the objective was moved in 100-nm steps from −3 to +3  μm relative to the sample focal plane. This corresponds to 61 positions for each of which an image was recorded. Images were taken without and with the SPP in place. The SPP was in a standard nosepiece holder from Olympus that fit directly under the exit of the objective, which could easily be slid into or out of place.

Bead samples were illuminated by an LED with a peak wavelength of 470 nm (Thorlabs model 470L4). The detector was a Hamamatsu ORCA Fusion sCMOS camera with exposure set to 200 ms. Standard procedure was to select a region of interest (ROI) of 500×500  pixels ($54\times 54\;\mu m^{2}$), containing 4 to 20 beads. The LED intensity was adjusted to fill the intensity range of the 16-bit camera. The maximum intensity occurred in the middle plane (focal plane depth within the slide) where the hotspot of the RPSF pattern was most compact and most intense. The fluorescent beads had SNRs at the focal plane of ∼100 and at extreme depth detunings of ±3  μm, the SNR was 28. Both SNR values were calculated using the maximum intensity of the hotspot compared with nearby background levels. The SNR values for cell samples, on the other hand, were 27 and 19, respectively, for the first and last frames in the time series, where photobleaching is the cause of the decreasing fluorescent intensity with time.

Ten replicates of the same field of view (FOV) were collected to improve the SNR of the images. These replicates were automatically collected using micromanager and Pycro-Manager software to control the motion along z of the stage as well as the camera image recording. A total of 11 FOV’s representing 153 distinct beads were analyzed.

A similar setup, based on a motorized Olympus IX-83, was used for live cell imaging. Position in x/y was controlled using an ultrasonic motorized stage (Olympus BX3-SSU), whereas the z position was controlled using the motorized nosepiece, enabling collection of z-stacks with 100-nm steps over a 6-μm range, as for the bead samples. Sample drift was avoided using the z-drift compensation system (Olympus IX3-ZDC). Cells were kept in physiological conditions with a stage-top incubator (Tokai Hit), maintaining 37°C and 5% ${\mathrm{CO}}_{2}$. Images were recorded with a Hamamatsu ORCA Flash 4.0 camera, controlled by the CellSens software (Olympus).

### 2.3 Sample with Fluorescent Beads

We used an Olympus objective designed specifically to match the optical environment of a cell. Thus, our fluorescent bead sample was optically designed to match fluorescent emitters inside a cell. To achieve this, the sample consisted of a microscope slide coated with a flat plane of 100-nm-diameter yellow-green fluorescent polystyrene microspheres (Fluoresbrite YG microspheres, Polysciences Inc, Warrington, Pennsylvania, United States) embedded in a plane between two layers of optical adhesive (Norland Products #NOA 139, Jamesburg, New Jersey, United States), whose refractive index, 1.39, closely matched the optical environment of cells.

### 2.4 Mammalian Cells

U2OS osteosarcoma cells with a stable genomic integration of Lactose repressor (LacR) binding arrays (256 copies of Lactose operon - LacO^35^) were used to track chromatin motion in 3D. The cells were seeded in 35-mm glass-bottom dishes (MatTek) at 100,000 cells per dish and maintained in Dulbecco’s modified Eagle medium (DMEM) supplemented with 10% FBS (Sigma) in a humidified incubator set to 37°C, 5% ${\mathrm{CO}}_{2}$. To visualize the LacO repeats, LacR fused to green fluorescent protein - GFP (LacR-GFP) was transiently expressed by transfection of the corresponding plasmid DNA using Lipofectamine 3000 (Invitrogen), 24 hr before imaging. For fixation, the cells were incubated for 20 min in 4% paraformaldehyde solution (Sigma cat# HT5011).

### 2.5 Template Matching to Determine 3D Position from 2D Images

To recover the x, y, and z positions of the beads from the 2D camera images, we used a library of 61 images (templates), each being the image of a single bead. The images were acquired with the SPP in place. The z position of the beads (defocus) fell between −3 and +3  μm about the plane of Gaussian focus. The template images were 51×51  pixels with the bead centered in each image. For each of the 10 image sets in a FOV, a template bead was selected to create the template library. The template bead was the bead that produced the most symmetric pattern for the average intensity z-projection of all 61 planes. A study of many beads in multiple FOVs indicated that this optimal point of minimal spherical aberration was located about 5  μm to the left and 10  μm above the actual center pixel of the camera FOV. Planes were collected so that plane #1 was at or close to δz=−3  μm, and plane #61 was close to δz=+3  μm.

Template matching was applied in two stages. In the first stage, we used a built-in MATLAB function, normxcorr2, that computed the intensity-based normalized 2D cross-correlations between each template image and each target image. This function translates the “moving” target image one pixel at a time in both x and y directions. The criterion for best match is the maximum in the 2D cross-correlation value. This method is limited to pixel precision, ±0.5  pixel, or about 54 nm, in x and y. In the second stage, we used paraboloid surface fit technique^36^ to find x and y to subpixel precision. For the determination of z, the second stage consisted of using a simple one-dimensional (1D) quadratic curve fit to the three points around the optimal template for a given target plane. In this way we could find the z-axis localization to subpixel precision.

### 2.6 Tracking Fluorescently Labeled DNA Regions in Live Cells

The template-matching approach (Sec. 2.5) was used on live cells as in the case of fluorescent beads. A template library of images was generated from 61 planes taken for a fluorescently labeled Lac array in a fixed cell. The Olympus IX-83 motorized stage was moved in 100-nm increments over a 6-μm range while collecting fluorescent images. A 51×51  pixel^2^ ROI was created around the center of the fluorescent region in the cell nucleus. We also used the MOSAIC Particle Tracker (ImageJ plugin)^37^ to track the same fluorescently labeled DNA spots with the SPP removed, i.e., with no SPP. This allowed us to compare our 3D tracking results with classic 2D tracking.

### 2.7 Determining the Diffusion Coefficient from Tracking Data in Live Cells

Mean-squared displacement (MSD) values were computed for all time points (t) according to the following equation:

$$\mathrm{MSD}(\tau )=\langle {\left(\overset{\to }{r}(t+\tau )-\overset{\to }{r}(t)\right)}^{2}\rangle =2nD\tau ,$$ (2)

where the angle brackets ⟨…⟩ indicate an average over all time points, t, and r is the position vector as a function of time. Here n is the dimension of the space (n=3 for 3D tracking), and D is the diffusion coefficient. Diffusion coefficients were derived from the computed MSD values and Eq. (2).

### 2.8 Vector-Field Simulation of the RPSF for a Microscope with a Single-Spot Generating SPP

**Fig. 3:**
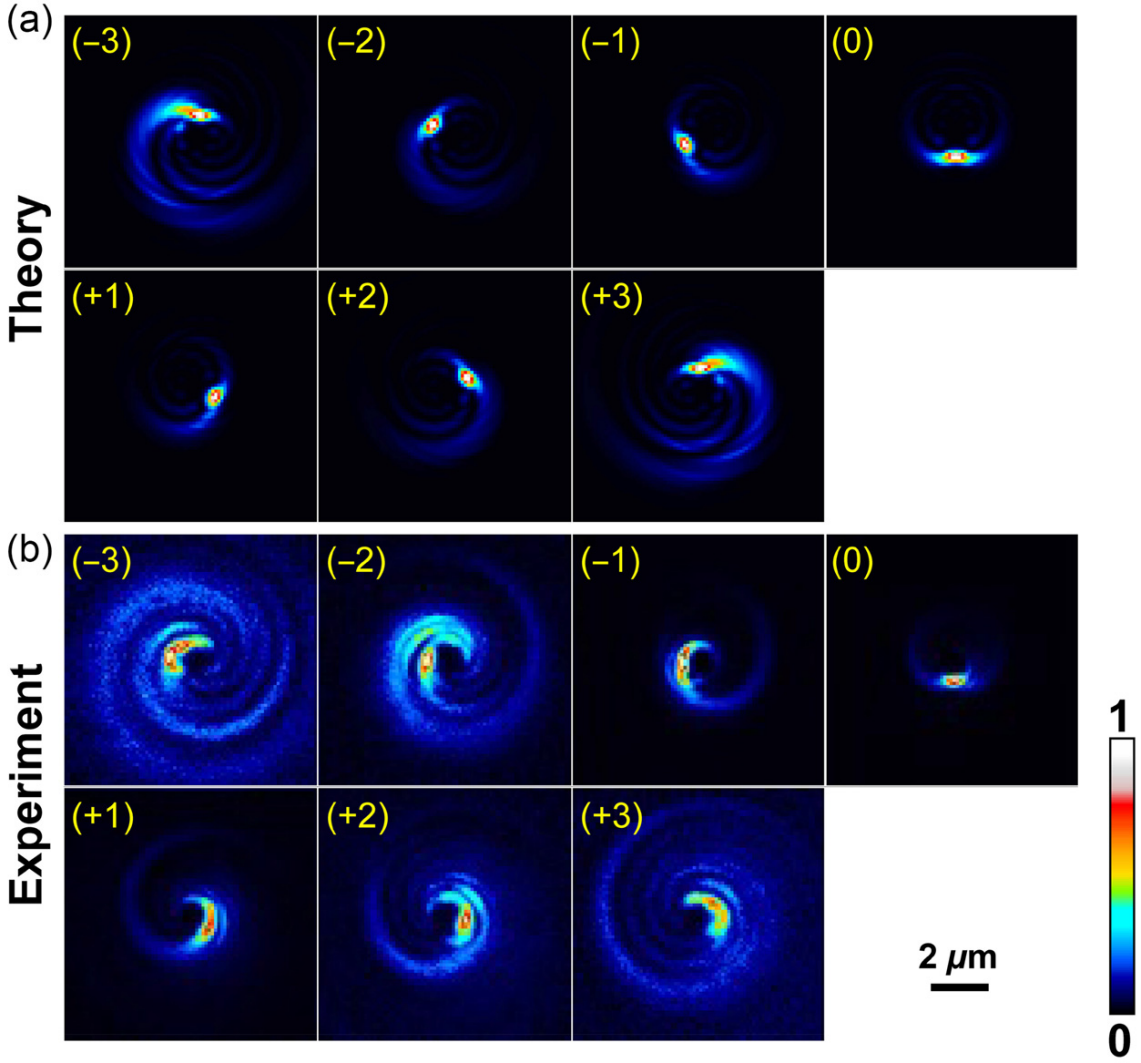
(a) Montage of theoretical spiral patterns, represented in false color (see the color bar for values of the image intensity), produced by the single-spot SPP both above and below the nominal focal plane for the same optical parameters as those used to collect experimental data. The value of the defocus $\delta z_{0}$, in μm, is indicated in parentheses in each image. (b) Montage of the corresponding experimental patterns. The maximum intensity in each sub-image in both montages is normalized to 1. Panels (a) and (b) are represented at the same scale, as they would appear projected into the sample.

Our vector field simulations of the rotating PSF were based on expressions in Ref. 9. The appropriate equations were translated into MATLAB code, which was used to generate the theoretical RPSFs shown in Fig. 3(a) and to make predictions about our experimental observations. The MATLAB code is available as a Supplementary Material in Code 1 (Ref. 38).

## 3 Results and Discussion

### 3.1 Optical Performance of the SPP

We recorded 61 RPSFs of a 100-nm-diameter spherical fluorescent bead with the source bead located in the range $\delta z_{0}=-3$ to +3  μm. The Gaussian focal plane is defined by $\delta z_{0}=0\;\mu m$. Figure 3(a) shows a montage of seven theoretical images of a 100-nm bead with z-gaps of 1  μm (total range −3 to +3  μm) generated by vector field calculations using the design parameters of the SPP with seven zones, as shown in Code 1 (Ref. 38). The beads were endowed with a Lambertian reflectance profile in our simulations. The length scale matches the scale expected under the experimental conditions of the microscope system. Intensities are normalized to 1 for each subimage. Normalized experimental sub-images collected for the same seven planes are shown in Fig. 3(b). Similarities between the two sets are the rotations of the maximum intensity regions with depth, the creation of spiral arms of growing width as depth detuning increases, and the reversal of helicity of the spiral pattern as one goes from negative detunings (z<0  μm) to positive detunings (z>0  μm) and vice versa. There are also qualitative differences between the calculated and observed “spiral galaxies” outside the “hot spot,” including a broader, more diffuse footprint of the experimental images at larger distances from focus.

There are several key quantitative metrics to characterize the optical behavior of the SPP. The original goal motivating the design was to produce a single spot rotating on a circle with the angle of rotation depending linearly on depth. The selection of the zones (i.e., the number, type, and area of each zone of the SPP) was based on these criteria.

**Fig. 4:**
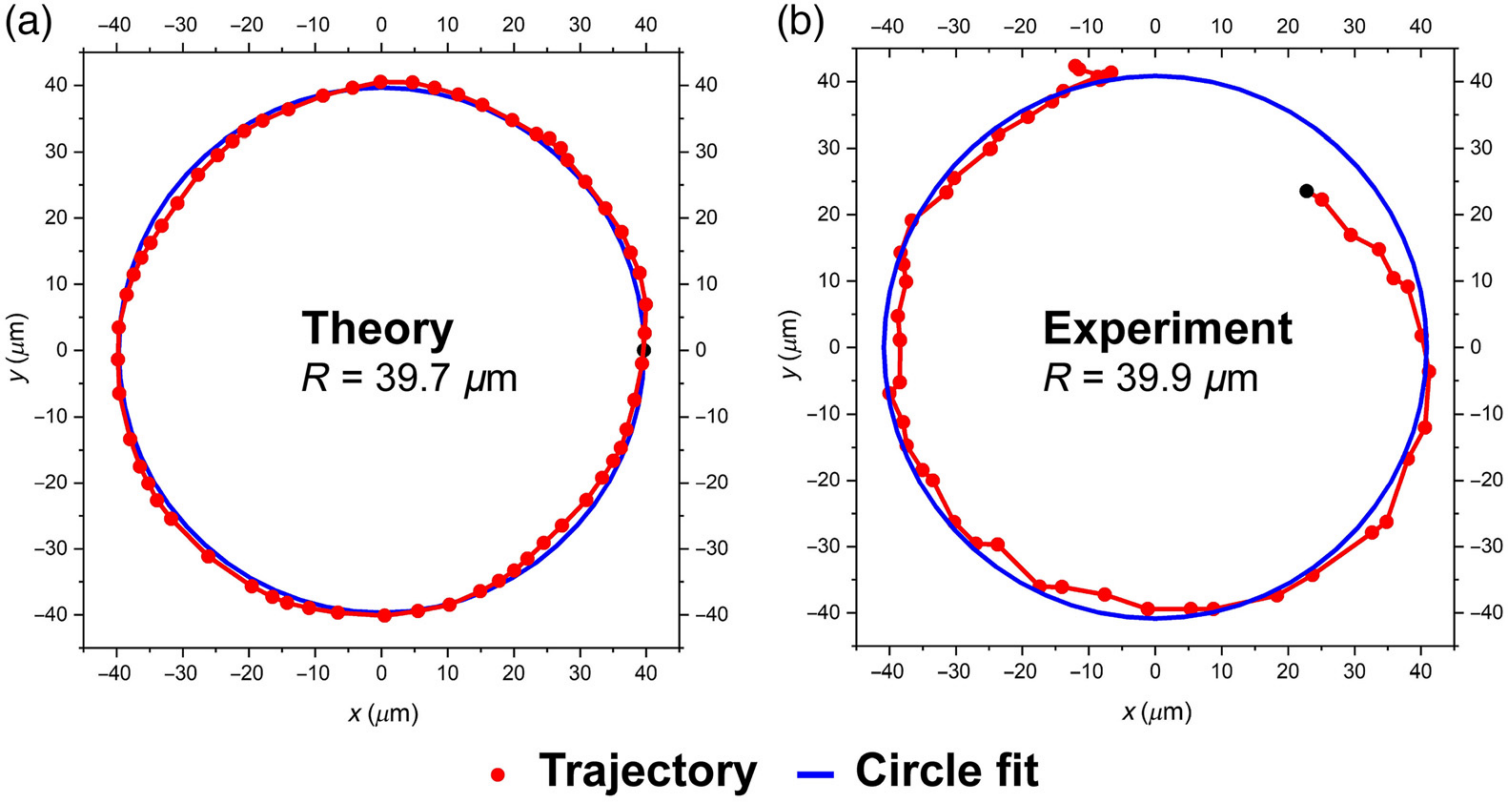
Hotspot trajectories: theory versus experiment. (a) Theoretically calculated hotspots from a set of 51 images of the irradiance pattern corresponding to depths in the range −2 to +3  μm in intervals of δz=100  nm. (b) Corresponding experimental measurements. Also shown (in blue) is the best fit circle to the hotspot data. The first point at z=−2  μm is indicated by a black dot. Scale is that in the camera plane.

As the plane is moved along z, the hotspot from a bead rotates in a circle about the z axis (Figs. 1 and 3). The bright spot ideally moves along a circle of fixed radius as the detuning from focus changes. We identified the centers of the bright spots at each position, using a center of mass intensity algorithm. Figure 4(a) shows the trajectory of the intensity peaks in the theoretical images [like those shown in Fig. 3(a)] for a range of depths corresponding to −2 to +3  μm in increments of 100 nm. The range from −3 to −2  μm is not shown because most of the experimental datasets in this range had their spots split into two angularly separated spots, preventing us from determining spot center positions. With the design of the SPP, the theoretical points from −2 to +3  μm fill an angular range that is about 1.2 radians (∼70  deg) >2π. In Fig. 4(b), we show corresponding experimental data with the same range of depths and increments. The 51 measurements follow the theory well. The circle fits to these trajectory plots allow us to compare the experimental results to theory. The predicted radius of the trajectory circle in the camera plane was 39.7  μm. The observed radius for the specific bead in Fig 4(b) was 39.9  μm. The measured radius from the combined data on 56 beads was 39.1±1.4  μm [mean ± standard deviation (SD)]. The theoretical and experimental values for the circle radius differ by 2%.

Another important quantitative metric is the design criterion of having a uniform ROR, i.e., the hotspot should rotate on a circle such that the angle versus z-depth plot is linear. An approximate analytical expression for the rotation rate of the bright spot in the intensity distribution produced by a point emitter is given in Eq. (1). This analytical rotation rate depends on several physical parameters, such as the objective NA=1.3, the center wavelength of the emitter, $\lambda_{0}=525\;\mathrm{nm}$, the largest OAM quantum imparted to photons by the SPP (L=7), and the refractive index of the medium, n=1.406. Substituting these values into Eq. (1) gives the theoretically predicted result of dθ/dz=1.49  rads/μm. Correspondingly, an axial range of 2π/1.49=4.2  μm is indicated for one complete rotation of the pattern around the center, which underestimates the experimentally observed range, as seen in Fig. 4 and which we discuss further near the end of the present subsection.

**Fig. 5:**
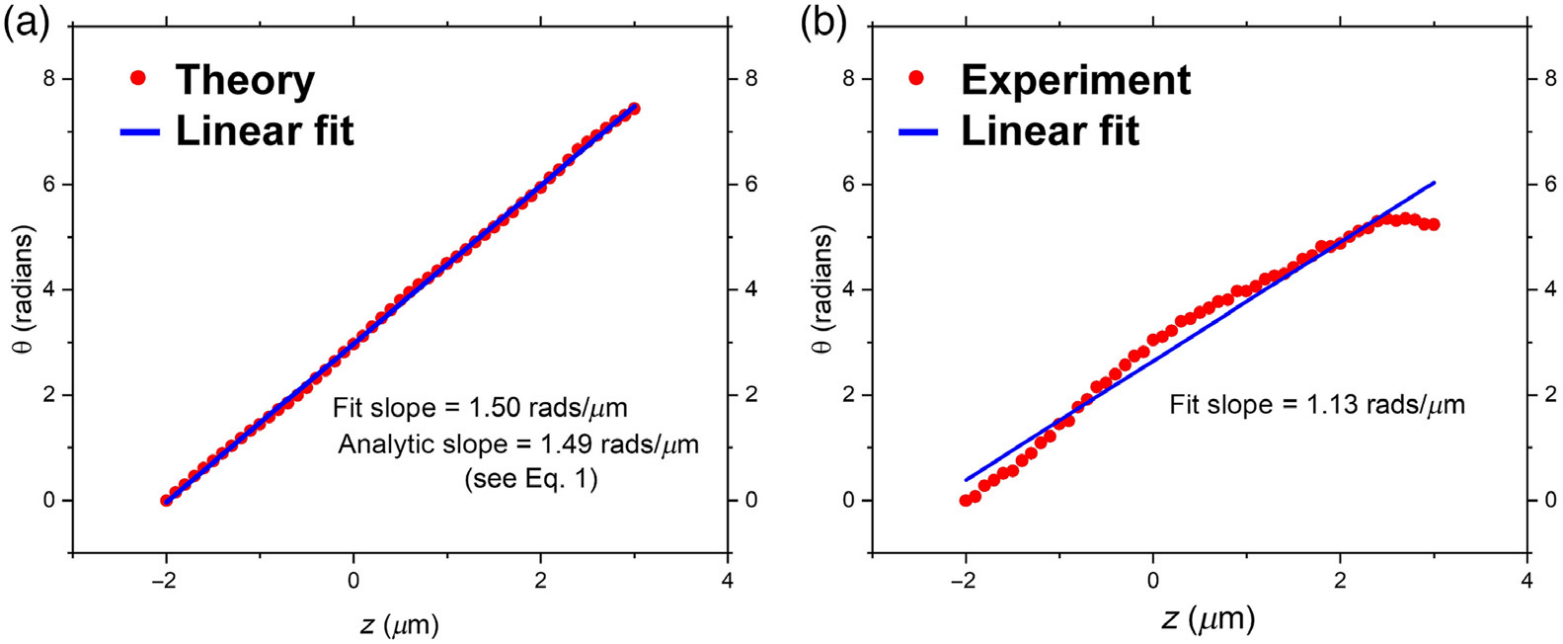
Hotspot rotation versus source depth: theory versus experiment. (a) Angles versus depth along z of theoretically calculated hotspots from a set of 51 images of the irradiance pattern corresponding to depths in the range −2 to +3  μm in intervals of δz=100  nm. Changes of rotation angle taken from the points in Fig. 4(a) with the zero of angle arbitrarily taken to be the angle at the point $\delta z_{0}=-2\;\mu m$. (b) Corresponding angles of experimentally observed hotspots (red circles) for the bead shown in Fig. 4(b). The hotspot rotates clockwise with increasing z as clearly seen from Figs. 3 and 4. The blue line is the best fit line.

To obtain a “theoretical” value for the expected rotation rate under high NA conditions, we processed the images under our exact high NA conditions (NA=1.3) and then selected the coordinates corresponding to the highest intensity pixel in the computed image. We plotted the data for the 51 points in Fig. 4(a) and found the slope by performing a linear fit to the data [Fig. 5(a)]. Here the red circles correspond to the maximum values of the points in the theoretically generated images for the different depths. The blue curve is the best linear fit to the red circles and gives a resulting theoretical slope of dθ/dz=1.50  rads/μm, in excellent agreement with the analytical formula given in Eq. (1).

The corresponding experimental plot of angle versus z-depth is shown in Fig. 5(b) and this is taken from the trajectory plotted in Fig. 4(b). The red circles correspond to the center-of-mass of intensity values that equal or exceed 0.9 of the maximum intensity. Figure 5(b) shows the experimental data for the template bead in a FOV with four beads. Here we show the same range as in Fig. 4(b), namely $\delta z_{0}=-2$ to +3  μm. For the same set of data that we used to report the trajectory radius (trajectories of 56 beads in 56 FOVs distributed over 2 samples), we found that ⟨dθ/dz⟩=0.99±0.08  rads/μm (mean ± SD). This experimental ROR is 34% lower than the value given by the analytical expression, Eq. (1). Note that in Fig. 5(b), the slope corresponds to that of a single bead in a single FOV, so its slope differs from the mean value above that corresponds to 56 beads in different FOVs.

One possible explanation for the discrepancy between the predicted and observed RORs is that since the SPP is not exactly at the exit pupil plane (EPP) of the objective, we would need to correct for their separation. Equivalently, one must map the SPP back to that plane. For rays diverging when incident on the SPP, which is the case in our microscope, with the SPP placed downstream in the turret nosepiece, this would mean that the SPP would appear both demagnified and diffractively blurred when mapped back to the EPP of the objective. A demagnified SPP is equivalent to a larger effective L (note the zone radii are inversely proportional to the square root of L for low NA with a more complicated decrease with L for high NA). Changing effective L from 7 to about 10 would bring the theoretically predicted ROR into agreement with the experimentally observed ROR and the theoretical maximum axial range for one complete rotation, over which the pattern deteriorates unacceptably, into agreement with the observed range of about 6  μm.

The diffractive blurring of the SPP when mapped back to the objective EPP, on the other hand, is possibly responsible for the second important difference between the theoretically predicted and observed bead images, namely the broader, more diffuse footprint of the latter. Note, however, that despite an imperfect SS-RPSF design spurred by our desire to achieve simplicity of implementation, we still successfully tracked the diffraction-limited spots with this DOE in this configuration with good localization precision for DNA loci in live cells, as discussed in Sec. 3.3.

### 3.2 SPP Localization Performance

To assess SS-RPSF localization performance, for each FOV we used a 59×59  pixel^2^ ROI around the template bead. Template matching was performed in a two-stage process that first used the 2D cross-correlation function in MATLAB to determine which template image best matched each of the 61 z-plane ROI target images for each bead in the FOV. This template-matching method only used translation along x and y to find the best template to match a given bead’s ROI z-plane image. The minimum transverse translation step was 1 pixel.

For stage two, we found the location in the lateral dimensions to subpixel precision using a quadratic surface fitting procedure that used a 3×3  pixel region around the location of maximum 2D cross correlation and fit a general paraboloid (allowing both elliptic and hyperbolic paraboloids) to estimate where the cross-correlation maximum occurred between integer pixel shifts of the image and template. A nonlinear least-squares fitting function in MATLAB was used to generate the best fitting parameters, namely the x-y location that gave the maximum cross-correlation.

To locate the cross-correlation maximum along z to subpixel precision, we fitted a parabola to the 3 values of z below, at, and above the best fit template plane. The vertex of that parabola was determined analytically. In both the transverse and axial cases, we also computed the standard deviation of the localization differences between adjacent z-steps. This is a metric for uncertainty in the precision of changes in location during tracking.

Overall, this simple two-step cross-correlation template-matching approach with only translational degrees of freedom gave reasonably precise localization results, as described below in detail. Our goal was to meet or exceed the localization/tracking precision achieved in our earlier work in 2D where we localized and tracked chromatin microdomains with a precision of about 40 nm.^39^ Here we show that the mean transverse localization precisions along x and y, as they differ from ground truth, were $\langle \sigma_{x}\rangle =21\pm 13\;\mathrm{nm}$ and $\langle \sigma_{y}\rangle =24\pm 6\;\mathrm{nm}$, (mean ± SD) over the entire 6-μm depth range; the mean axial localization precision difference from ground truth was $\langle \sigma_{z}\rangle =70\pm 15\;\mathrm{nm}$ over the 6-μm depth range. Considering a smaller depth range (2.9  μm), the localization precision values are: $\langle \sigma_{x}\rangle =19\pm 10\;\mathrm{nm}$, $\langle \sigma_{y}\rangle =24\pm 4\;\mathrm{nm}$, and $\langle \sigma_{z}\rangle =50\pm 3\;\mathrm{nm}$ (Fig. 7). These results with fluorescent latex spheres match or exceed our previous 2D results for transverse localization of fluorescent chromatin microdomains in cells. Localization in the axial direction is slightly less precise, as expected. This data are based on beads that were at least 4.32  μm (40 camera pixels) from their nearest neighbor and included 77,410 bead images.

Our x, y, and z precisions are also comparable with those achieved by other state-of-the-art 3D localization techniques such as 3D STochastic Optical Reconstruction Microscopy (STORM),^13^ double-spot PSFs,^3^^,^^11^ and phase-ramp PSF,^14^ but our results extend over an axial depth range that is 1.5 to 3 times larger, with a simple low-cost retrofitting of a commercially available microscope. Although some more recent approaches can achieve even better z precisions that are comparable to the x, y precisions of order 20 nm, they either do so over a much smaller axial range or require highly sophisticated optical-bench setups. The use of supercritical-angle fluorescence^40^ can generate such high z precision but only over a small sample thickness of order 150-nm contiguous to the cover slip, while rotating PSF interferometric imaging^41^ can do so over 2- to 3-μm depths but only by means of an expensive dual-opposed-objective layout.

Most recently, deep-learning methods, such as DeepSTORM3D,^42^ have exhibited the potential to increase the localization precision further, even with spatially highly extended PSFs, such as overlapping tetrapod PSFs.^7^ Deep-learning methods have also been successfully applied to optimize paired PSFs to achieve precise 3D localization at high densities.^43^ Similar machine-learning approaches with the single-spot RPSF such as that employed here can also potentially improve its error performance even under lower SNR and higher emitter-density conditions.

#### 3.2.1 Localization agreement between ground truth and experiment (metric #1)

The key metric for evaluating how well particle motion can be tracked in a movie is the correct determination of the change in position from one frame to the next, i.e., an accurate measure of the change in position between adjacent frames.

##### Transverse motion

**Fig. 6:**
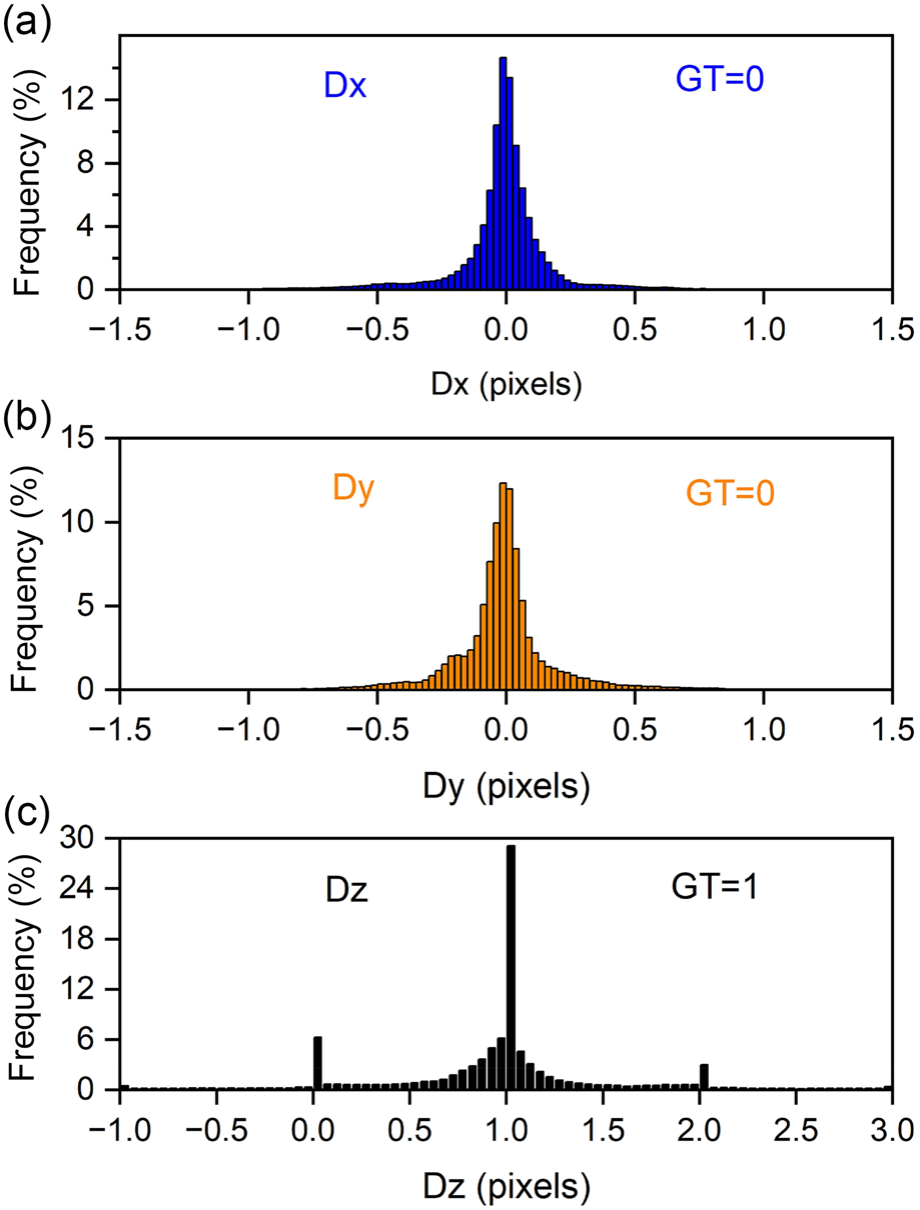
Localization agreement between ground truth and experiment. (a) Histogram of the percentage of bead images whose change in x-axis location agrees (Dx=0  pixels per step, blue) or disagrees (Dx≠0  pixels per step) with ground truth, for axial steps. (b) Histogram of the percentage of bead images whose change in y-axis localization agrees (Dy=0  pixels, orange) or disagrees (Dy≠0  pixels) with ground truth, for axial steps in z. (c) Histogram of the percentage of bead images whose change in z-axis location agrees (Dz=1  pixel, black) or disagrees (Dz≠1  pixel) with ground truth, for axial steps in z.

In stepping along z, we did not intentionally move the sample along the transverse directions. Thus, we expect adjacent z-planes would have the same x, y values. We analyzed the motion to subpixel localization precision using the two-step process described above. Figures 6(a) and 6(b) show frequency (percentage) histograms of the subpixel localization precisions for motions along x and y, respectively, compared with the ground truth of no motion (change of 0 pixels in both x and y).

**Fig. 7:**
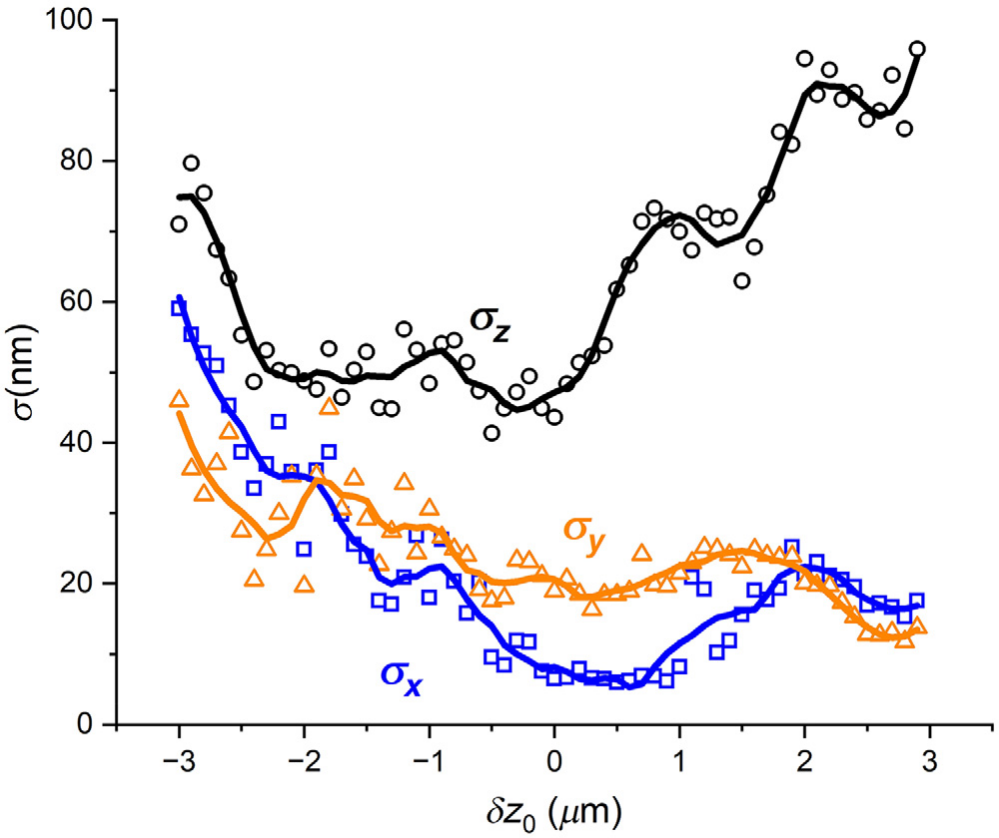
Localization precision (standard deviation – $\sigma_{x}$, $\sigma_{y}$, $\sigma_{z}$) for changes in x (blue open squares), y (orange open triangles), and z (black open circles) compared with ground truth as a function of the z-depth.

Note that the localization data includes all 61 planes for each bead in the FOV, including the 10 of the 61 planes that do not follow the theoretical trajectory shown in Fig. 4. Yet, as these results show, such beads can still be located with reasonable transverse localization precision and so the localizable z-depth range is the full 6-μm range over which we gathered data. This result meets our expectations qualitatively because the 10 frames at the larger depths have distinctive intensity distributions visually.

##### Axial motion

Because each bead was moving in 100-nm steps along z between successive images (for 61 planes), we expect that the difference in the template images that matched these would differ by 1 plane or 100 nm. Thus, we performed the same two-step template matching procedure described at the beginning of this section, which gave us an estimate of the subpixel localization precision. Figure 6(c) provides the histogram frequency data for the distribution of z-step values measured by template-matching, where the ground truth is that $\delta z_{0}=100\;\mathrm{nm}$ for each step. One pixel in Fig. 6(c) is 100 nm. Clearly there is a peak at a change in pixel of 1 in the distribution as expected. Also, to be expected are subsidiary peaks at nearby integer values (0 and 2), but with points in between due to the use of subpixel localization precision methods (parabolic fitting in 1D). This distribution differs significantly from a Gaussian due to its high peak at 1 as well as the subsidiary peaks at 0 and 2. We note that the high peak at one is due to the naturally larger number of points that would land at the intended change, but also note that it is partially due to assigning the subpixel fraction to 0 for cases where the second step of fitting a quadratic function to the three planes (with the template-identified plane in the middle) created a result that indicated a move of one or more pixels. We assigned these cases a fractional subpixel shift of 0 since we do not allow a subpixel-determining operation to shift our final position by one pixel or more.

An estimate for the z-axis localization precision is given in the next section where we separately analyze the localization precision for the 60 different steps between the 61 planes through the whole range of depths.

#### 3.2.2 Localization precision versus z-depth ($\delta z_{0}$) (metric #2)

In Fig. 3 the experimental intensity patterns appear to deviate from theory more the farther the sources are detuned from the focal plane. Thus, it is useful to characterize how the localization precision depends on the z-depth detuning from the focal plane. Figure 7 presents the results of analyzing all three localization precisions (x, y, and z) at a given z-plane or depth for all beads in all FOVs and in all sets.

The data show that the localization precision depends on the detuning from the focal plane. For example, the transverse localization precision along x, $\sigma_{x}$, for the plane at $\delta z_{0}=-3\;\mu m$. is significantly worse (about 8×) than the $\sigma_{x}$ of the focal plane at $\delta z_{0}=0\;\mu m$. In the case of the axial localization precision, $\sigma_{z}$, the plane at −3  μm is about 1.6× worse than the focal plane, whereas the other edge of the range, at +3  μm, the localization precision is about 2.1× worse than at the focal plane. Overall, the data provide useful information on the precision that might be achieved when tracking objects along the z direction using this single-spot SPP coupled with a template-matching algorithm. The mean localization precision values over all the planes in the three plots are: $\langle \sigma_{x}\rangle =21\pm 13\;\mathrm{nm}$, $\langle \sigma_{y}\rangle =24\pm 6\;\mathrm{nm}$, and $\langle \sigma_{z}\rangle =70\pm 15\;\mathrm{nm}$, (mean ± SD). The lowest localization precision values for z-values occur in the region of $\delta z_{0}$ ranging from −2.5 to +0.4  μm (2.9  μm range) and result in localization precision values of $\langle \sigma_{x}\rangle =19\pm 10\;\mathrm{nm}$, $\langle \sigma_{y}\rangle =24\pm 4\;\mathrm{nm}$, and $\langle \sigma_{z}\rangle =50\pm 3\;\mathrm{nm}$. In the work by Roider et al.,^11^ based on a cross-polarization scheme to generate a two-spot pattern from the earliest, low-NA SPP design of Ref. 8, the best localization precision achieved was 25 nm. For comparison, the DH-PSF system^3^ reported an axial localization precision of single fluorescent molecules of 20 nm over a range of 2  μm. Our result over the 2.9  μm range where $\langle \sigma_{z}\rangle =50\;\mathrm{nm}$ is good, given the compromises made to achieve lower cost and simplicity.

The z-mapping precision may be further improved with single-spot RPSF data by deconvolving with the ADMM algorithm followed by Taylor expansion for subpixel resolution.^44^ This has been shown to work well with DS-RPSF data. Higher precision z-mapping may also be achievable by using machine learning (with a library of single spot SPP-derived images of point emitters with known z-position as ground truth). Also, higher precision may be achievable experimentally by using an SPP with a lower value of L—see Eq. (1)—which should increase the ROR for a smaller range of z distances, thereby increasing the precision along z.

### 3.3 Tracking DNA Loci in Live Cells

**Fig. 8:**
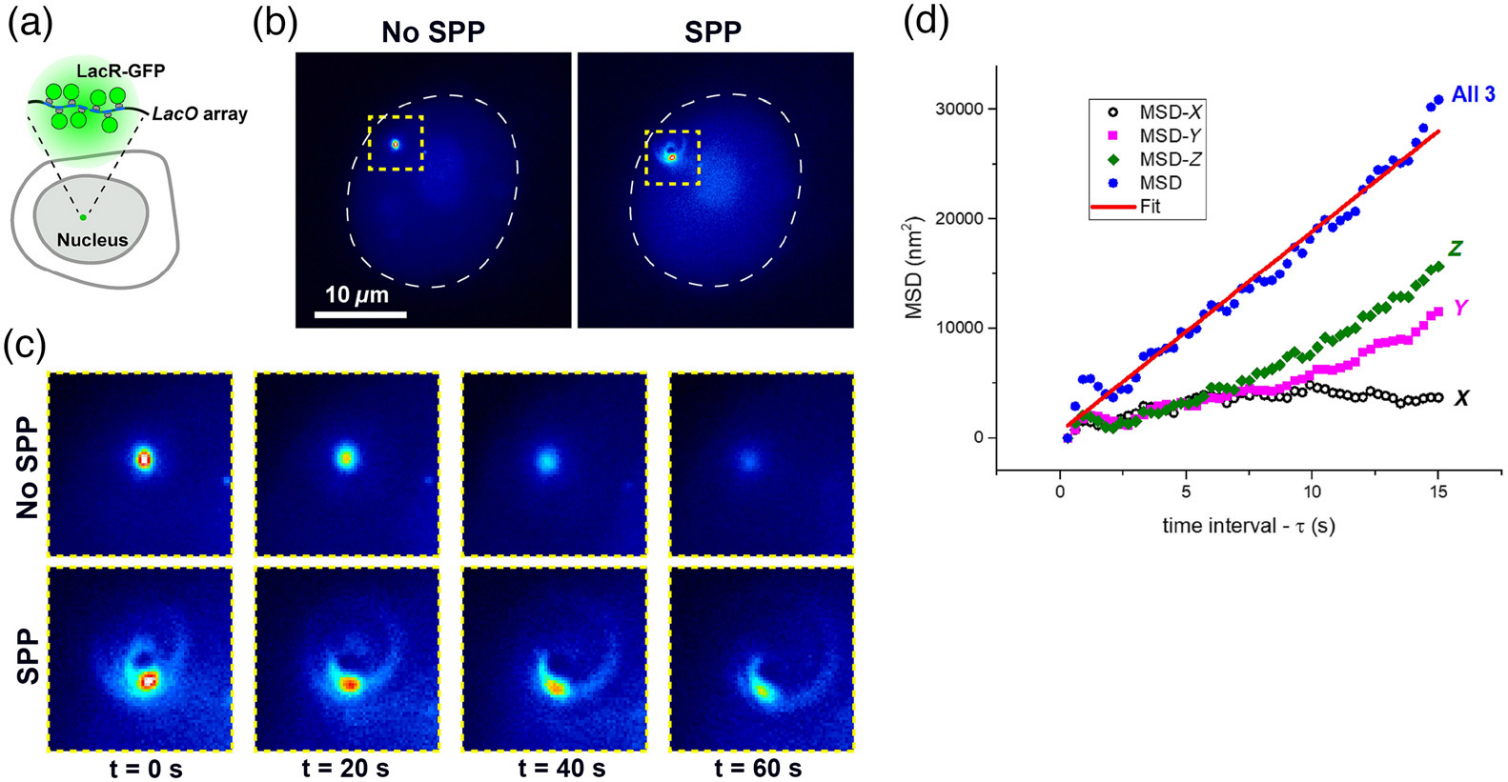
(a) Schematic of a cell with a genomic integration of LacO DNA arrays and expressing LacR-GFP to visualize these arrays. (b) Image of a LacR-GFP fluorescent spot within the nucleus (boundary highlighted with white dotted line). Images were taken without or with SPP. (c) Enlargement of the regions delineated with yellow dotted lines in panel (b) and displayed at different time points. (d) Plot of the MSD versus time for the spot shown in panel (a). MSD for all three dimensions (blue solid circles), MSD along z (green diamonds), MSD along y (magenta squares), and MSD along x (black open circles).

**Fig. 9:**
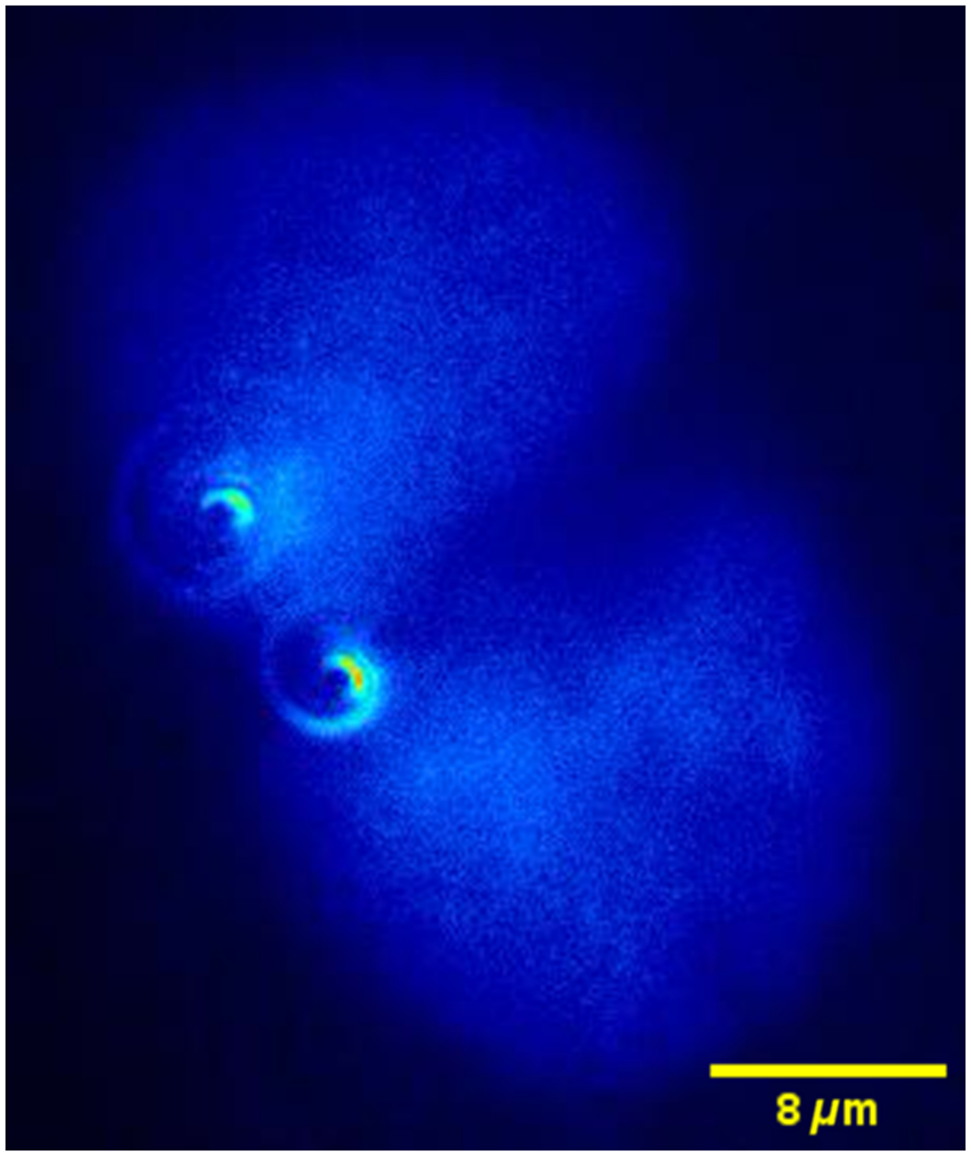
Two LacR-GFP DNA spots in different U2OS cell nuclei imaged as a function of z-depth. The two spots are at different depths in the sample (Video 1, MPEG, 126 KB [URL: https://doi.org/10.1117/1.JBO.27.12.126501.s1]).

We expressed the LacR-GFP and a nuclear localization signal in U2OS cells with a stable genomic integration of LacO arrays to fluorescently label subdiffraction spots of chromatin in cell nuclei [Fig. 8(a)]. This method is well established to study chromatin dynamics in yeasts and mammalian cells. LacR-GFP signals could easily be imaged using the SPP [Figs. 8(b) and 8(c)]. First, a library of cell images was generated, using fixed cells with LacR-GFP signals, moving the objective by 100-nm steps, as for the fluorescent beads sample (Fig. 9). This library was used for template-matching as described above. Note that the template library generated from fixed cell data is used for all subsequent live-cell data analyses. There was no need to generate new template libraries for each cell or biological replicate. Next, we tracked LacR-GFP spots for 60 s (300 ms exposure for 200 frames) in live cells. Figure 8(d) plots the MSD for a LacR-GFP trace, corresponding to the cell shown in Fig. 8(b). In addition to the overall MSD given by Eq. (2), we also plot the separate components (x/y/z) for comparison.

MSDs can be used to extract diffusion coefficients, D, which correspond to the fitted slope of the MSD versus τ plot divided by 6 [Eq. (2)]. Figure 8(d) shows the linear fit to the 3D motion data, which yielded a diffusion coefficient value of $D=301\;{\mathrm{nm}}^{2}/s$, in agreement with previous reports on chromatin diffusion.^45^^,^^46^ It also agrees with analysis of the 2D motion of the same LacR-GFP spot but tracked with the SPP removed (no SPP). In that case, we found that D (no SPP) = $380\;{\mathrm{nm}}^{2}/s$.

3D single-particle tracking of fluorescently labeled DNA subregions has been reported in live yeast cells by Moerner’s group using both a double-helix phase plate^47^ and a tetrapod phase plate.^48^ These previous studies demonstrate the important role 3D tracking can play to better understand chromatin dynamics in cell nuclei, which is the goal of our investigations. Both studies from the Moerner group involved tracking two GFP-labeled LacO/LacI foci in yeast chromosomes, near the Galactose (GAL) locus. Diffusion coefficients, derived from motions measured over 10 s (0.1-s intervals) were $∼2000\;{\mathrm{nm}}^{2}/s$. There are differences between the time scales in this previous study (10 s) and our data (60 s). Moreover, the nuclear environments of yeast and mammalian cells differ, further contributing to different measurement outcomes. In a more recent publication, the same group studied anomalous diffusion of chromatin regions in human cells.^24^ The DH-PSF was used to track chromatin loci in 3D, on fast (∼several minutes) and slow (hours) timescales. The effective lateral and axial chromatin diffusion values were ∼850 and $∼700\;{\mathrm{nm}}^{2}/s$, respectively, in the same range as those measured here. The differences are likely due, at least in part, to the fact that different genomic positions were tracked in the two studies.

## 4 Conclusions

We provide the first experimental evaluation of 3D localization microscopy using a single-spot rotating PSF based on Bessel beams. After passing through the SPP, the light beam’s phase front consists of a linear superposition of a number of “nondiffracting” Bessel beams. The fused silica SPP was placed near the back focal plane of the objective of an inverted microscope, using the Nomarski prism slider. A potential advantage of a single-spot PSF compared with multispot PSFs, such as the DH-PSF, is its lower risk of mismatching spot pairs when the emitters are in close proximity. Although unproven at this stage, such a comparison is the subject of future research in our system.

Another challenge in the current template-matching localization method, related to density, is the parameter of template size. A systematic study of how localization precision is affected by smaller templates will be carried out using a denser sample dataset we intend to collect in the future. To be able to use smaller templates, we also plan to use a numerical modeling approach to find a more optimal phase-plate pattern that tightens the resulting intensity patterns with depth. Other improvements that will be considered include corrections for “wobble,” which are depth-dependent lateral shifts in point images that arise from spherical aberrations.^49^

With well-separated beads, we obtained reasonable agreement when comparing the experimental versus theoretical diameter and rotation trajectory of the “hotspots” generated by the SPP in the image plane. Although the ROR did not agree precisely with the prediction of theory, our observations showed a large range where the ROR was uniform, a design criteria of the SPP. We achieved precise 3D localization using this simple system. The localization precision was best for lateral tracking with $\sigma_{x}≃\sigma_{y}≃23\;\mathrm{nm}$, but the axial-tracking precision was worse at $\sigma_{z}≃70\;\mathrm{nm}$. These values of standard deviations of the differences in estimated steps compared with ground truth steps are obtained by averaging those differences over the whole depth range of 6  μm, but axial precision can be improved to ∼50  nm if we limit localization to a smaller depth range of about 3  μm.

Finally, we 3D-tracked subdiffraction regions of DNA in live cells and extracted the diffusion coefficient for the motion. We have demonstrated the proof-of-principle for 3D-tracking with a simple system that makes use of single-spot images generated by the multivortex SPP. Our approach should be easily configurable for investigators who envision installing 3D-tracking capability into their own systems.

## 5 Code, Data, and Materials Availability

Code used to design the SPP and analyze the data used to generate the figures are accessible on the Open Science Framework (OSF) website.^38^ The data used to generate Figs. 3–7 are also available at the OSF site.
